# Supplementary material for: A Likelihood Approach for Real-Time Calibration of Stochastic Compartmental Epidemic Models
Source: PLoS Comput Biol. 2017 Jan 17;13(1):e1005257. doi: 10.1371/journal.pcbi.1005257 (PMC5240920; doi:10.1371/journal.pcbi.1005257)
Supplement: S1 File — (TAR.GZ) [file pcbi.1005257.s014.tar.gz › HSPH_Online-SI-Revision/PF08/n1-extreme/n1-extreme_PF2_table8.pdf]

1	newly 3 weeks spec	5.	10.7	{2., 21.}	135.2	0.372466	newly 3 weeks cumul	23.	43.14	{15., 85.}	103.043	0.290184	newly 5 weeks spec	0.1	6.9	{1., 15.}	6814.	1.61937	newly 5 weeks cumul	25.	59.13	{20., 117.}	147.64	0.357039	newly 52 weeks cumul	29.	84.67	{24., 166.}	203.828	0.411637
2	newly 3 weeks spec	4.	10.33	{1., 23.}	189.25	0.502873	newly 3 weeks cumul	26.	37.11	{8., 64.}	77.2692	0.278699	newly 5 weeks spec	4.	6.83	{0., 16.}	122.25	0.479744	newly 5 weeks cumul	34.	51.92	{11., 91.}	86.2941	0.299243	newly 48 weeks cumul	44.	82.8	{12., 152.}	123.091	0.357507
3	newly 3 weeks spec	13.	15.	{3., 29.}	61.3846	0.316984	newly 3 weeks cumul	58.	54.94	{23., 106.}	45.1724	0.23034	newly 5 weeks spec	7.	10.3	{1., 20.}	94.8571	0.424355	newly 5 weeks cumul	73.	77.7	{26., 137.}	51.3699	0.240413	newly 50 weeks cumul	89.	126.17	{35., 221.}	78.3708	0.292105
4	newly 3 weeks spec	10.	12.63	{3., 24.}	70.9	0.33051	newly 3 weeks cumul	62.	43.77	{12., 73.}	47.1129	0.31352	newly 5 weeks spec	6.	8.57	{0., 19.}	96.8333	0.459295	newly 5 weeks cumul	83.	63.03	{15., 107.}	48.3253	0.314027	newly 49 weeks cumul	123.	113.42	{19., 247.}	64.2114	0.372925
5	newly 3 weeks spec	9.	5.18	{1., 11.}	56.	0.526938	newly 3 weeks cumul	33.	19.75	{3., 40.}	51.7879	0.408074	newly 5 weeks spec	4.	4.23	{0., 10.}	81.75	0.56443	newly 5 weeks cumul	44.	28.8	{5., 55.}	50.5	0.386	newly 42 weeks cumul	59.	50.27	{8., 111.}	65.3729	0.423549
6	newly 3 weeks spec	11.	12.8	{3., 26.}	70.9091	0.338426	newly 3 weeks cumul	43.	41.95	{11., 85.}	53.9767	0.283434	newly 5 weeks spec	6.	9.4	{1., 21.}	111.333	0.465653	newly 5 weeks cumul	53.	62.93	{13., 127.}	66.0566	0.29217	newly 47 weeks cumul	71.	131.84	{23., 351.}	127.239	0.372195
7	newly 3 weeks spec	6.	12.12	{3., 22.}	122.667	0.352759	newly 3 weeks cumul	35.	44.	{14., 81.}	57.8857	0.222681	newly 5 weeks spec	5.	8.56	{1., 19.}	111.6	0.408032	newly 5 weeks cumul	52.	62.72	{22., 116.}	57.	0.233866	newly 51 weeks cumul	64.	104.36	{27., 215.}	92.7188	0.290018
8	newly 3 weeks spec	9.	6.54	{0., 14.}	61.5556	0.536853	newly 3 weeks cumul	27.	23.68	{3., 49.}	54.2963	0.394995	newly 5 weeks spec	1.	4.79	{0., 11.}	425.	0.704423	newly 5 weeks cumul	32.	33.74	{5., 70.}	65.5	0.402091	newly 45 weeks cumul	35.	59.06	{5., 156.}	118.057	0.45506
9	newly 3 weeks spec	3.	13.85	{4., 26.}	367.	0.584008	newly 3 weeks cumul	16.	52.87	{20., 88.}	232.313	0.444588	newly 5 weeks spec	3.	9.27	{1., 20.}	234.333	0.485005	newly 5 weeks cumul	22.	73.39	{26., 122.}	235.773	0.443404	newly 52 weeks cumul	25.	109.97	{33., 209.}	341.64	0.529987
10	newly 3 weeks spec	17.	6.85	{1., 17.}	65.8235	0.709144	newly 3 weeks cumul	46.	24.05	{3., 55.}	58.8043	0.5657	newly 5 weeks spec	5.	4.42	{0., 12.}	85.2	0.706064	newly 5 weeks cumul	57.	33.76	{4., 79.}	61.5088	0.568065	newly 45 weeks cumul	77.	60.63	{4., 149.}	75.7013	0.607906
11	newly 3 weeks spec	5.	10.59	{3., 25.}	135.	0.370998	newly 3 weeks cumul	17.	39.43	{16., 80.}	139.588	0.319602	newly 5 weeks spec	0.1	6.93	{1., 15.}	6846.	1.59141	newly 5 weeks cumul	19.	55.21	{20., 117.}	196.263	0.395972	newly 51 weeks cumul	19.	84.48	{21., 189.}	348.632	0.540555
12	newly 3 weeks spec	1.	13.66	{4., 24.}	1272.	1.05932	newly 3 weeks cumul	17.	52.99	{19., 82.}	219.118	0.475946	newly 5 weeks spec	2.	9.69	{2., 19.}	400.5	0.644509	newly 5 weeks cumul	22.	74.11	{23., 122.}	245.682	0.509464	newly 52 weeks cumul	22.	124.44	{29., 253.}	473.909	0.663683
13	newly 3 weeks spec	20.	6.57	{0., 14.}	67.85	0.7697	newly 3 weeks cumul	80.	23.2	{2., 47.}	71.	0.760492	newly 5 weeks spec	17.	4.46	{0., 10.}	74.2353	0.927144	newly 5 weeks cumul	116.	33.42	{3., 69.}	71.1897	0.786423	newly 45 weeks cumul	196.	60.53	{3., 139.}	70.1582	0.838472
14	newly 3 weeks spec	2.	8.21	{1., 17.}	333.5	0.587375	newly 3 weeks cumul	9.	31.13	{8., 59.}	255.889	0.466373	newly 5 weeks spec	0.1	5.71	{0., 12.}	5652.	1.32324	newly 5 weeks cumul	9.	43.76	{10., 76.}	394.667	0.585944	newly 49 weeks cumul	9.	72.02	{13., 144.}	708.	0.720178
15	newly 3 weeks spec	2.	6.88	{0., 16.}	281.	0.637699	newly 3 weeks cumul	21.	23.58	{3., 53.}	75.3333	0.45669	newly 5 weeks spec	1.	4.58	{0., 11.}	408.	0.745795	newly 5 weeks cumul	25.	33.49	{3., 72.}	90.2	0.46671	newly 44 weeks cumul	29.	58.17	{3., 125.}	148.31	0.535579
16	newly 3 weeks spec	13.	7.55	{0., 16.}	59.3077	0.645906	newly 3 weeks cumul	45.	28.69	{2., 57.}	55.8889	0.512072	newly 5 weeks spec	6.	5.47	{0., 12.}	74.8333	0.614973	newly 5 weeks cumul	59.	40.17	{2., 82.}	56.9322	0.519955	newly 51 weeks cumul	82.	65.75	{2., 167.}	66.0122	0.566276
17	newly 3 weeks spec	14.	11.3	{1., 25.}	58.	0.449733	newly 3 weeks cumul	63.	40.42	{10., 83.}	50.8571	0.375718	newly 5 weeks spec	8.	7.26	{0., 17.}	69.5	0.516048	newly 5 weeks cumul	82.	56.75	{13., 112.}	51.5	0.374181	newly 48 weeks cumul	132.	102.43	{16., 247.}	66.3712	0.453868
18	newly 3 weeks spec	15.	12.76	{3., 23.}	50.	0.363667	newly 3 weeks cumul	37.	47.33	{12., 83.}	61.1622	0.244458	newly 5 weeks spec	5.	8.53	{1., 17.}	110.6	0.432383	newly 5 weeks cumul	50.	66.32	{19., 119.}	66.32	0.262031	newly 51 weeks cumul	60.	103.23	{24., 229.}	102.75	0.325115
19	newly 3 weeks spec	10.	14.96	{3., 27.}	81.	0.304117	newly 3 weeks cumul	28.	55.38	{19., 92.}	110.929	0.301802	newly 5 weeks spec	4.	11.16	{2., 25.}	202.	0.441137	newly 5 weeks cumul	43.	79.44	{26., 145.}	102.14	0.292237	newly 52 weeks cumul	65.	132.	{33., 255.}	127.723	0.341211
20	newly 3 weeks spec	4.	4.7	{0., 10.}	89.5	0.516051	newly 3 weeks cumul	15.	19.75	{3., 40.}	78.4667	0.353569	newly 5 weeks spec	2.	2.88	{0., 7.}	124.	0.640402	newly 5 weeks cumul	21.	26.52	{4., 59.}	77.8095	0.366244	newly 45 weeks cumul	30.	44.63	{5., 116.}	108.833	0.433643
21	newly 3 weeks spec	20.	11.61	{1., 24.}	55.75	0.54075	newly 3 weeks cumul	78.	40.06	{6., 79.}	55.1795	0.49049	newly 5 weeks spec	5.	8.37	{0., 21.}	124.2	0.527827	newly 5 weeks cumul	98.	57.7	{6., 117.}	53.7959	0.47274	newly 48 weeks cumul	119.	102.81	{7., 244.}	64.6975	0.472707
22	newly 3 weeks spec	4.	9.96	{1., 20.}	177.5	0.458965	newly 3 weeks cumul	28.	36.36	{9., 68.}	70.9286	0.279621	newly 5 weeks spec	5.	6.81	{1., 15.}	98.2	0.468123	newly 5 weeks cumul	41.	50.88	{12., 95.}	70.	0.287884	newly 49 weeks cumul	59.	83.48	{15., 146.}	94.1356	0.350111
23	newly 3 weeks spec	8.	14.64	{3., 33.}	113.5	0.315792	newly 3 weeks cumul	27.	53.	{17., 113.}	114.741	0.28907	newly 5 weeks spec	5.	10.67	{2., 25.}	141.8	0.380382	newly 5 weeks cumul	38.	76.36	{25., 157.}	120.263	0.297115	newly 51 weeks cumul	59.	129.65	{29., 309.}	147.644	0.343524
24	newly 3 weeks spec	5.	10.19	{2., 21.}	135.	0.400658	newly 3 weeks cumul	19.	38.22	{13., 69.}	117.579	0.320226	newly 5 weeks spec	2.	5.96	{0., 15.}	233.	0.553158	newly 5 weeks cumul	25.	51.83	{18., 95.}	124.12	0.330331	newly 52 weeks cumul	25.	73.14	{20., 147.}	206.48	0.429278
25	newly 3 weeks spec	8.	14.32	{3., 27.}	106.75	0.349473	newly 3 weeks cumul	31.	49.19	{17., 88.}	82.4839	0.270532	newly 5 weeks spec	8.	10.26	{1., 20.}	85.	0.445699	newly 5 weeks cumul	48.	70.79	{18., 140.}	76.3125	0.271181	newly 49 weeks cumul	114.	123.51	{26., 239.}	64.7105	0.312781
26	newly 3 weeks spec	4.	9.79	{2., 18.}	165.25	0.429199	newly 3 weeks cumul	13.	37.01	{14., 67.}	190.538	0.413425	newly 5 weeks spec	1.	5.29	{0., 13.}	459.	0.699625	newly 5 weeks cumul	14.	49.66	{18., 98.}	259.429	0.491083	newly 51 weeks cumul	19.	69.79	{21., 147.}	273.421	0.481314
27	newly 3 weeks spec	15.	7.82	{0., 18.}	60.2667	0.634539	newly 3 weeks cumul	54.	28.03	{4., 62.}	57.9815	0.528235	newly 5 weeks spec	8.	5.68	{0., 16.}	69.75	0.61142	newly 5 weeks cumul	70.	41.2	{6., 94.}	59.1714	0.524641	newly 46 weeks cumul	110.	72.	{6., 178.}	66.7273	0.579811
28	newly 3 weeks spec	9.	11.61	{2., 26.}	79.8889	0.369131	newly 3 weeks cumul	43.	40.46	{11., 74.}	53.0233	0.290121	newly 5 weeks spec	3.	7.88	{0., 20.}	192.	0.507376	newly 5 weeks cumul	53.	57.25	{14., 109.}	60.283	0.294012	newly 50 weeks cumul	70.	92.12	{15., 205.}	82.0571	0.341565
29	newly 3 weeks spec	2.	7.91	{1., 16.}	316.5	0.604479	newly 3 weeks cumul	14.	30.61	{8., 56.}	135.929	0.371625	newly 5 weeks spec	0.1	5.52	{0., 13.}	5450.	1.43818	newly 5 weeks cumul	14.	42.86	{11., 84.}	217.571	0.470289	newly 47 weeks cumul	17.	66.5	{14., 135.}	303.882	0.539851
30	newly 3 weeks spec	2.	4.49	{0., 11.}	181.5	0.594826	newly 3 weeks cumul	16.	15.94	{1., 35.}	74.5	0.516455	newly 5 weeks spec	3.	3.46	{0., 9.}	112.667	0.757756	newly 5 weeks cumul	21.	23.26	{1., 54.}	85.2381	0.556111	newly 42 weeks cumul	31.	39.6	{1., 104.}	108.065	0.638966
31	newly 3 weeks spec	4.	12.19	{2., 25.}	224.25	0.462553	newly 3 weeks cumul	13.	45.04	{15., 79.}	250.154	0.466768	newly 5 weeks spec	3.	7.63	{1., 16.}	183.667	0.448297	newly 5 weeks cumul	20.	62.44	{18., 105.}	218.5	0.420658	newly 51 weeks cumul	27.	93.67	{25., 165.}	254.259	0.435614
32	newly 3 weeks spec	1.	12.04	{1., 25.}	1110.	0.96293	newly 3 weeks cumul	8.	44.37	{16., 88.}	458.125	0.67286	newly 5 weeks spec	0.1	7.34	{0., 18.}	7268.	1.54773	newly 5 weeks cumul	8.	61.64	{16., 125.}	673.25	0.794991	newly 51 weeks cumul	10.	99.76	{26., 230.}	901.2	0.855645
33	newly 3 weeks spec	21.	9.64	{1., 21.}	57.2381	0.570438	newly 3 weeks cumul	67.	36.72	{7., 71.}	51.1045	0.425964	newly 5 weeks spec	11.	7.06	{1., 16.}	57.8182	0.539034	newly 5 weeks cumul	89.	51.63	{11., 105.}	50.1011	0.411481	newly 47 weeks cumul	124.	91.65	{15., 200.}	57.7823	0.437709
34	newly 3 weeks spec	9.	11.78	{2., 24.}	72.	0.317752	newly 3 weeks cumul	31.	43.48	{14., 78.}	68.2581	0.243692	newly 5 weeks spec	2.	8.17	{0., 18.}	339.5	0.662023	newly 5 weeks cumul	36.	61.85	{16., 111.}	94.4167	0.284243	newly 50 weeks cumul	40.	104.03	{22., 193.}	179.475	0.383057
35	newly 3 weeks spec	11.	14.02	{3., 26.}	76.	0.332491	newly 3 weeks cumul	46.	49.33	{15., 84.}	52.6739	0.238889	newly 5 weeks spec	5.	10.42	{1., 22.}	149.6	0.462447	newly 5 weeks cumul	59.	71.96	{19., 129.}	64.4068	0.264536	newly 51 weeks cumul	68.	131.22	{25., 298.}	127.912	0.350144
36	newly 3 weeks spec	10.	11.59	{2., 24.}	68.9	0.352373	newly 3 weeks cumul	26.	43.08	{12., 84.}	93.5385	0.289811	newly 5 weeks spec	7.	8.62	{0., 18.}	82.8571	0.447343	newly 5 weeks cumul	37.	62.02	{15., 119.}	96.7568	0.300586	newly 48 weeks cumul	53.	100.85	{18., 241.}	122.094	0.353254
37	newly 3 weeks spec	11.	5.18	{0., 12.}	60.7273	0.643066	newly 3 weeks cumul	35.	18.31	{3., 41.}	55.4	0.50511	newly 5 weeks spec	7.	3.51	{0., 8.}	62.7143	0.737713	newly 5 weeks cumul	50.	25.99	{3., 56.}	55.22	0.51126	newly 43 weeks cumul	55.	46.07	{4., 107.}	70.2364	0.50746
38	newly 3 weeks spec	6.	10.14	{1., 22.}	104.667	0.403002	newly 3 weeks cumul	20.	37.96	{11., 78.}	112.	0.327796	newly 5 weeks spec	2.	7.29	{0., 15.}	305.5	0.679421	newly 5 weeks cumul	28.	54.33	{12., 111.}	119.893	0.354585	newly 48 weeks cumul	40.	91.7	{13., 196.}	157.9	0.405824
39	newly 3 weeks spec	6.	14.45	{3., 31.}	158.5	0.375805	newly 3 weeks cumul	22.	53.12	{18., 101.}	151.273	0.340397	newly 5 weeks spec	2.	10.1	{1., 23.}	427.	0.635669	newly 5 weeks cumul	28.	75.17	{22., 152.}	177.679	0.371457	newly 51 weeks cumul	34.	125.44	{28., 337.}	278.059	0.460895
40	newly 3 weeks spec	20.	5.79	{0., 14.}	75.35	0.967841	newly 3 weeks cumul	45.	22.07	{1., 49.}	63.9333	0.652867	newly 5 weeks spec	5.	4.33	{0., 10.}	81.8	0.691751	newly 5 weeks cumul	56.	31.28	{2., 70.}	63.4643	0.630411	newly 49 weeks cumul	62.	55.14	{2., 117.}	77.4839	0.595316
41	newly 3 weeks spec	17.	6.87	{0., 16.}	63.	0.696	newly 3 weeks cumul	35.	25.17	{4., 49.}	51.9143	0.430468	newly 5 weeks spec	9.	5.55	{0., 13.}	64.3333	0.74477	newly 5 weeks cumul	50.	36.71	{5., 70.}	51.86	0.452824	newly 44 weeks cumul	61.	65.78	{6., 146.}	77.6066	0.512149
42	newly 3 weeks spec	1.	12.72	{3., 28.}	1172.	0.980044	newly 3 weeks cumul	20.	48.14	{17., 88.}	147.4	0.327523	newly 5 weeks spec	1.	8.78	{1., 20.}	786.	0.795074	newly 5 weeks cumul	23.	67.54	{23., 145.}	197.217	0.386286	newly 52 weeks cumul	25.	99.6	{28., 215.}	300.48	0.469522
43	newly 3 weeks spec	13.	15.42	{4., 30.}	65.8462	0.283447	newly 3 weeks cumul	49.	54.19	{17., 101.}	52.4286	0.230329	newly 5 weeks spec	1.	10.82	{2., 23.}	986.	0.895833	newly 5 weeks cumul	57.	77.36	{24., 148.}	68.4912	0.24585	newly 51 weeks cumul	68.	131.56	{31., 266.}	118.765	0.309944
44	newly 3 weeks spec	6.	9.91	{1., 20.}	109.167	0.419106	newly 3 weeks cumul	30.	37.92	{8., 76.}	72.0667	0.291819	newly 5 weeks spec	3.	6.33	{0., 16.}	158.333	0.511103	newly 5 weeks cumul	42.	52.23	{10., 101.}	73.5952	0.304208	newly 49 weeks cumul	55.	85.36	{13., 169.}	104.	0.359261
45	newly 3 weeks spec	9.	13.71	{3., 28.}	85.4444	0.316891	newly 3 weeks cumul	41.	50.72	{18., 92.}	60.9268	0.249946	newly 5 weeks spec	11.	8.82	{2., 18.}	53.0909	0.359108	newly 5 weeks cumul	64.	70.05	{22., 129.}	53.3281	0.246803	newly 52 weeks cumul	78.	106.61	{28., 215.}	75.8077	0.279625
46	newly 3 weeks spec	12.	12.13	{2., 23.}	59.75	0.337739	newly 3 weeks cumul	35.	45.62	{11., 82.}	68.	0.26347	newly 5 weeks spec	5.	7.88	{1., 18.}	115.6	0.502073	newly 5 weeks cumul	52.	63.47	{15., 117.}	66.4038	0.278026	newly 49 weeks cumul	69.	101.11	{19., 213.}	92.1884	0.333198
47	newly 3 weeks spec	16.	15.89	{4., 31.}	58.1875	0.346006	newly 3 weeks cumul	65.	55.01	{16., 107.}	49.3385	0.287193	newly 5 weeks spec	11.	10.65	{1., 23.}	69.9091	0.464076	newly 5 weeks cumul	87.	78.67	{22., 155.}	53.3218	0.296057	newly 50 weeks cumul	118.	138.38	{32., 307.}	78.8983	0.349479
48	newly 3 weeks spec	17.	16.69	{4., 36.}	65.7059	0.340219	newly 3 weeks cumul	49.	53.43	{17., 117.}	62.5102	0.270332	newly 5 weeks spec	7.	12.32	{1., 31.}	120.857	0.426629	newly 5 weeks cumul	64.	79.74	{26., 164.}	71.375	0.274293	newly 50 weeks cumul	85.	158.57	{35., 361.}	125.306	0.35221
49	newly 3 weeks spec	8.	9.56	{3., 18.}	62.25	0.265029	newly 3 weeks cumul	31.	37.51	{15., 69.}	53.4516	0.207795	newly 5 weeks spec	3.	6.31	{1., 13.}	145.	0.434639	newly 5 weeks cumul	39.	51.22	{19., 93.}	61.3846	0.222317	newly 52 weeks cumul	53.	75.6	{26., 141.}	78.0755	0.256789
50	newly 3 weeks spec	6.	7.26	{0., 18.}	88.6667	0.527562	newly 3 weeks cumul	38.	25.64	{2., 59.}	58.2105	0.512989	newly 5 weeks spec	6.	5.2	{0., 13.}	73.	0.631461	newly 5 weeks cumul	47.	36.64	{2., 79.}	60.5106	0.501622	newly 50 weeks cumul	63.	68.43	{2., 141.}	86.2063	0.551439
